# Graphene Oxide/RhPTH(1-34)/Polylactide Composite Nanofibrous Scaffold for Bone Tissue Engineering

**DOI:** 10.3390/ijms24065799

**Published:** 2023-03-18

**Authors:** Fan Fei, Haiyan Yao, Yujiang Wang, Junchao Wei

**Affiliations:** 1 School of Stomatology, Nanchang University, Nanchang 330006, China; 2 Jiangxi Province Key Laboratory of Oral Biomedicine, Nanchang 330006, China; 3 School of Chemistry and Chemical Engineering, Nanchang University, Nanchang 330031, China; 4 Jiangxi Province Clinical Research Center for Oral Disease, Nanchang 330006, China

**Keywords:** Polylactide, electrospinning, composite material, bone tissue engineering

## Abstract

Polylactide (PLA) is one of the most promising polymers that has been widely used for the repair of damaged tissues due to its biocompatibility and biodegradability. PLA composites with multiple properties, such as mechanical properties and osteogenesis, have been widely investigated. Herein, PLA/graphene oxide (GO)/parathyroid hormone (rhPTH(1-34)) nanofiber membranes were prepared using a solution electrospinning method. The tensile strength of the PLA/GO/rhPTH(1-34) membranes was 2.64 MPa, nearly 110% higher than that of a pure PLA sample (1.26 MPa). The biocompatibility and osteogenic differentiation test demonstrated that the addition of GO did not markedly affect the biocompatibility of PLA, and the alkaline phosphatase activity of PLA/GO/rhPTH(1-34) membranes was about 2.3-times that of PLA. These results imply that the PLA/GO/rhPTH(1-34) composite membrane may be a candidate material for bone tissue engineering.

## 1. Introductions

Bone is a dynamic tissue with the ability of self-reconstruction, repair and regeneration; it needs to be remodeled constantly to regulate bone homeostasis [1,2]. Nevertheless, congenital and acquired lesions, including trauma, infection, tumors and failed arthroplasty, may cause critical-size bone defects that the body cannot heal [3]. Bone tissue engineering (BTE) is an exciting strategy for regenerating bone defects that has attracted much attention. Biodegradable polymers acting as functional scaffolds have been critical factors in bone tissue engineering [4,5]. Polylactic acid (PLA) is a kind of aliphatic polyester commonly used in different biomedical fields, including bone and maxillofacial regeneration in the field of tissue engineering scaffolds, because of its good biocompatibility and biodegradability [6,7,8].

Various processes have been designed to produce functional polymer composites; traditional methods of preparing polymer fibers include melt spinning, solution spinning, liquid-crystal spinning and gel-spinning, across which the fiber diameters range generally in the micron level. Electrospinning technology is a popular and easy method of generating non-woven fibrous materials, by which bioactive materials can be added into a PLA matrix [9,10]. The fibrous membranes with a large surface area, high porosity and similar morphology to an extracellular matrix were formed under a high electric field to realize the rapid prototyping of nanofibers [8,9,10]. These are the advantages of electrospinning technology. However, the use of pure PLA electrospinning fibers in the field of bone regeneration often has some disadvantages, such as poor mechanical properties and low osteogenic induction activity, which cannot meet the requirements of clinical bone formation [11,12]. Therefore, the construction of PLA-based nanocomposites using electrospinning to blend PLA and other functional materials has attracted great attention, as this can combine the advantages of various materials and overcome the defects of single materials [10,13]. For example, composite electrospun fibers were fabricated by incorporating multi-wall carbon nanotubes (MWCNTs) and polyethylene glycol (PEG) into PLA, which effectively enhanced mechanical properties and accelerated osteogenic differentiation of stem cells [10,14].

Graphene oxide (GO) is a kind of intricate nanomaterial, possessing good biocompatibility and desirable chemical and physical properties, such as a high specific surface area and mechanical properties [15,16]. The structure of GO has many polar functional groups, such as hydroxyl and carboxyl groups, which can enhance the interactions between GO and the polymer matrix [17,18]. Therefore, GO is a promising nanofiller that may improve the mechanical properties of polymers. For example, the tensile strength of a GO/PVA nanofiber with 0.02 and 0.04 wt.% GO is 9.37 and 14.39 MPa, respectively, which is much higher than that of pure PVA (0.22 MPa) [18]. The content of GO has a critical effect on the mechanical properties of polymer composites. When GO is incorporated into PVA, if the concentration is lower than 1 wt.%, the tensile strength and elastic modulus will increase with the content; however, when the concentration increases to over 3 wt.%, the mechanical properties will decrease [19]. Many studies have proved that a low concentration of GO can promote cell adhesion and proliferation [15,17] and support the growth and osteogenic differentiation of stem cells in the field of bone tissue engineering [20]. GO has also had an overall significant positive effect on both in vitro differentiation and in vivo bone cell recruitment in the subcutaneous region [21]. A total of 0.5 wt.% GO was incorporated into fish gelatin/chitosan/genipin scaffolds, which enhanced the expression of runx2 and opn during osteogenic differentiation [21]. Therefore, a good way to improve the mechanical and osteogenic properties of polymers is the incorporation of GO as a nanofiller.

Parathyroid hormone (PTH) is a peptide hormone secreted by the parathyroid gland and has been approved by the US Food and Drug Administration (FDA) to treat bone diseases [22,23]. It can regulate calcium and phosphorus metabolism, promote osteoblast activity and accelerate bone transformation [22,24]. In contrast with other bone-promoting peptides, including BMP-derived peptides, osteogenic growth peptides and glucagon-like peptides, PTH is characterized by its dual role in promoting bone formation and stimulating bone resorption in the process of bone repair, which is highly similar to the process of bone formation under physiological conditions [25]. Recombinant human parathyroid hormone, which is named rhPTH(1-34), is a 1-34 amino acid fragment of the parathyroid hormone. In some animal experiments, it has been proved that a low-dose and intermittent administration of rhPTH(1-34) can promote bone regeneration and increase bone density by regulating RANKL/OPG ratios, whereas high-dose and continuous infusion can lead to bone absorption [22,23,24,26]. RhPTH(1-34) can also be combined with other materials. This has a better effect on osteogenesis than using rhPTH(1-34) alone. For example, hydrogels containing PTH(1-34) and nano-hydroxyapatite (nHAP) promoted cranial bone regeneration [27,28]. RhPTH(1-34) can cooperate with GO easily through electrospinning to fabricate composite scaffolds, which can promote osteogenic differentiation and bone regeneration. Furthermore, the impact of different rhPTH(1-34) stimulation modes on MC3T3-E1 cell proliferation and osteogenesis-related gene expression has been reported, and the expression levels of alkaline phosphatase and Runx2 were the highest in 10^−9^ M rhPTH(1-34) [29].

In this study, GO powder was dispersed in a polylactic acid solution, and then mixed with an rhPTH (1-34) solution to form a stable water-in-oil (w/o) emulsion to prepare biological composite fiber membranes via the electrospinning method. The results indicated that low concentrations of GO and rhPTH(1-34) can be successfully incorporated into PLA, and the composite membranes can improve aspects relevant for bone regeneration and biomedical applications, such as mechanical properties and synergistic osteogenic properties.

## 2. Results and Discussion

### 2.1. Characterization of Different Samples

**Figure 1:**
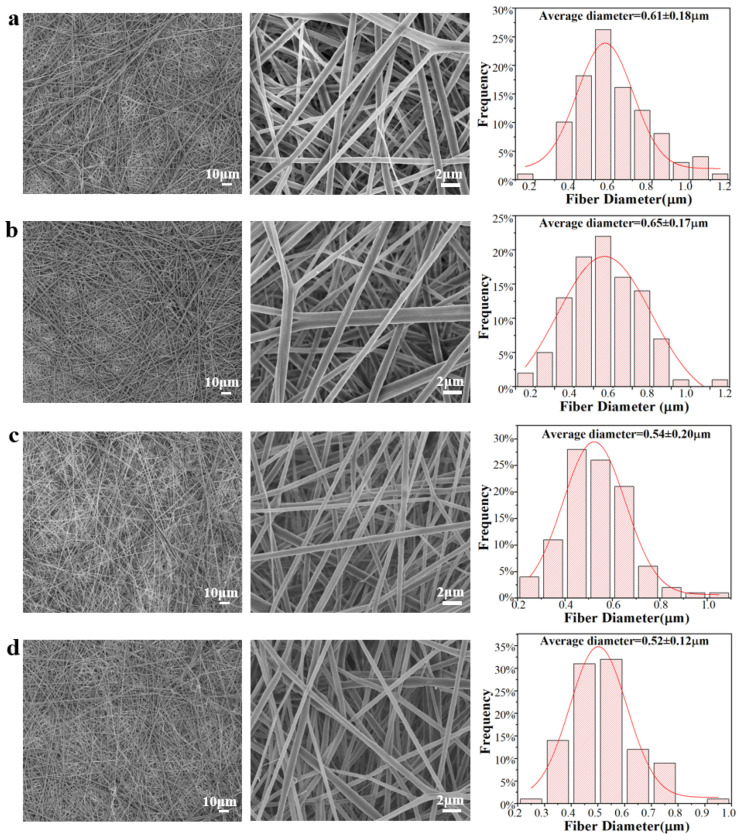
SEM images at different magnifications (500×, 5000×) and diameter distribution of electrospun membranes: (**a**) PLA; (**b**) PLA/rhPTH(1-34); (**c**) PLA/GO; (**d**) PLA/GO/rhPTH(1-34).

SEM of different electrospun membranes was employed to demonstrate the successful scaffolds prepared using the electrospinning hybrid method (Figure 1). Figure 1a,b shows the formed PLA and PLA/rhPTH (1-34) microfibers and the histogram graph of their microfibers, which were 0.61 ± 0.18 µm and 0.65 ± 0.17 µm, respectively. However, after adding 0.5 wt.% GO, the diameters of the PLA/GO and PLA/GO/rhPTH (1-34) microfibers decreased to 0.54 ± 0.2 and 0.52 ± 0.12 µm, respectively (Figure 1c,d). All microfibers were generally continuous and uniform, with a circular cross-section and porous surface morphology, and almost without any beaded structures. There was a slight difference between the surface morphology of the blends, the diameter values of microfibers decreased with the addition of GO, the fibers became more compact and the pore walls became rougher. The fibers were thinner because GO is electrically conductive, and the conductivity of the solution increased the charge density of the droplets that form on the tip of the needle, which helped the nanofibers to elongate and separate into thinner fibers [30,31]. When GO particles were added to the PLA emulsion, the crystallization of the solvent was modified, and the growth process of the crystals changed [32]. These results indicated that all the electrospun membranes had porous micrometer-level fiber networks with large surface area to volume ratios. The structure of the electrospun nanofiber network mimicked the natural structure of the natural extracellular matrix (ECM), which provides a natural basis for cell activities such as adhesion, proliferation, migration and metabolism, and plays a crucial role in cell function [33,34]. In bone tissue engineering, porous scaffolds serve as artificial ECMs to provide structural and mechanical support for the attachment, diffusion, propagation and differentiation of osteocytes, thus serving as cell growth templates for bone tissue regeneration [35].

**Figure 2:**
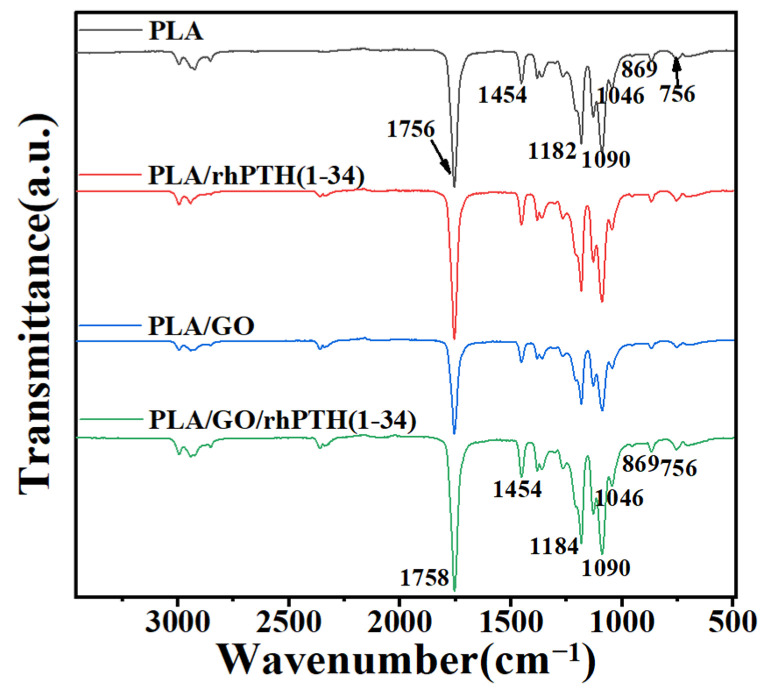
Fourier transform infrared (FTIR) spectra of PLA, PLA/rhPTH(1-34), PLA/GO and PLA/GO/rhPTH(1-34).

The Fourier transform infrared spectroscopy analysis of PLA, PLA/rhPTH(1-34), PLA/GO and PLA/GO/rhPTH(1-34) microfibers is shown in Figure 2. For PLA microfibers, the absorption bands appeared at 1756 cm^−1^ due to the –C=O stretching vibrations and at 1360 and 1454 cm^−1^ due to the bending and deformation vibrations of –CH_3_ bonds, respectively. 1182 cm^−1^ was due to the C–O–C stretching vibrations absorption peak, and 1090 cm^−1^ was due to the C–O bond deformation vibrations [36,37]. Because the experiments were performed using electrospinning technology for the preparation of membranes, GO and rhPTH (1-34) were added to the blends, and it was seen that the characteristic absorption bands of PLA were highlighted in the FTIR spectra of all the samples. Therefore, it was difficult to assess the presence of rhPTH (1-34) or even the presence of GO in the obtained membranes, and only slight shifts were detected with the addition of low concentrations of GO or rhPTH (1-34) compared with pure PLA microfibers.

**Figure 3:**
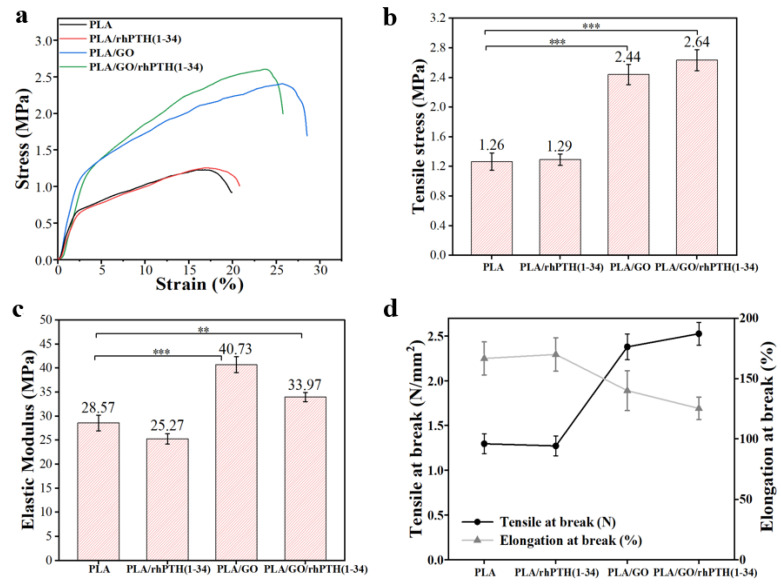
Tensile behavior of PLA, PLA/rhPTH(1-34), PLA/GO and PLA/GO/rhPTH(1-34) electrospun membranes. (**a**) Typical stress-strain curves; (**b**) tensile stress (Notes: *** *p* < 0.001); (**c**) elastic modulus (Notes: *** *p* < 0.001, ** *p* < 0.01); (**d**) tensile strength and elongation percentage. The average values and error bars are marked.

Mechanical strength is one of the main indexes used to evaluate the performance of the scaffolds; therefore, stable and suitable mechanical properties are necessary for scaffolds [38]. The stress-strain curves of the as-prepared composite membranes are shown in Figure 3a, and their tensile strengths are shown in Figure 3b. The overall deformation behavior of PLA membranes was similar to that of PLA/rhPTH(1-34) membranes, and their tensile strengths were 1.26 ± 0.12 MPa and 1.29 ± 0.076 MPa, respectively. With the addition of 0.5 wt.% GO, the tensile strengths increased to 2.44 ± 0.14 MPa and 2.64 ± 0.14 MPa, respectively. A gradual improvement was observed in Young’s modulus with evenly dispersed GO loading. The Young’s moduli of PLA and PLA/rhPTH(1-34) were increased from 28.57 ± 1.64 MPa and 25.27 ± 1.09 MPa to 40.73 ± 1.65 MPa and 33.97 ± 0.95 MPa, respectively (Figure 3c). The reinforcement effect of GO was due to the strong interaction between the molecular chains of PLA and GO and the lower fiber diameter, which facilitated better orientation of nanofibers in the membranes and made the membranes stiffer and stronger [39]. 

The elongation percentages of different composite membranes are shown in Figure 3d. The percentage elongation of PLA was 166.75 ± 13.75%. With the addition of GO, the percentage elongation of PLA/GO/rhPTH(1-34) was decreased to 125.34 ± 9.28%. This is due to the hydrogen bonding interaction that improved the molecular cohesion between PLA and GO, resulting in the reduction of segmental mobility [30,39,40]. These results suggest that the electrostatic force may improve the interaction between the polylactic acid matrix and the GO strengthening nanofillers during the electrospinning process, and that the size effect in nanofibers may also contribute to the improvement of mechanical properties, which is a positive phenomenon. It was in line with the definition of scaffolds in bone tissue engineering, as the scaffolds should have high mechanical strength and stiffness [41,42]. However, limited by the mechanical properties of electrospun membranes, the strength of pure PLA membranes are characterized by a strength of about 1.0 MPa, because the width and thickness of the membranes are greatly related to the mechanical properties. If the mechanical properties need to be further increased, surface modifications may be involved by increasing the thickness of the membrane. In addition, the mechanical strength of membranes prepared using the electrospinning method are relatively similar to those in the literature that has been published [41].

**Figure 4:**
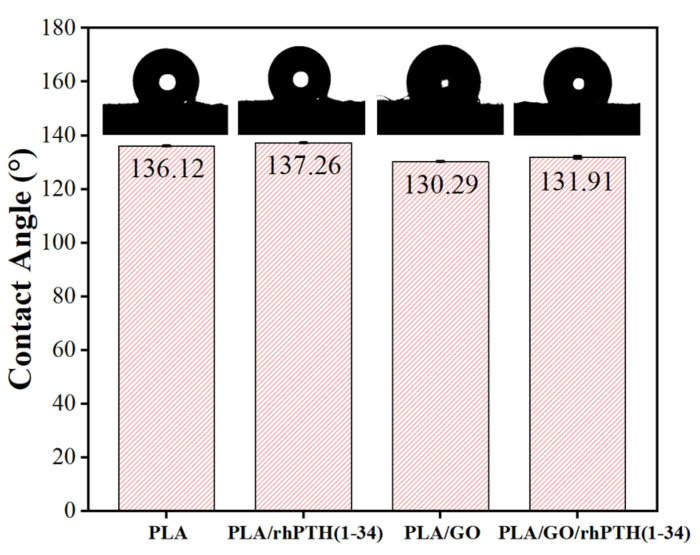
Water contact angle test results for different electrospun membranes. The contact angle is plotted as a function of composite membranes. The inset digital photos show the water droplets on the membranes (Notes: *p* > 0.05 stands for no significant difference).

The water contact angle with regard to the wettability of the electrospun membranes was measured because it has been reported that the improved hydrophilicity of a potential material intended for tissue engineering is favorable for cell proliferation [42,43]. Figure 4 shows that the contact angles of the pure PLA, PLA/rhPTH(1-34), PLA/GO and PLA/GO/rhPTH(1-34) membranes were 136.12°, 137.26°,130.29° and 131.91°, respectively. This result indicated that the differences between the contact angles of the four membranes were not obvious, and the additions of GO to hydrophobic polymers slightly improved the wettability of polymers. There are a large number of hydrophilic OH, C–O–C and COOH groups on the planes of carbon atoms in GO, which have good hydrophilicity and have been utilized to increase hydrophilicity and function [30,44,45]. During the electrospinning process, the GO was embedded inside of the nanofibers, resulting in only a small effect on the hydrophilicity of the composite membranes. The observation of this phenomenon might suggest a preferred interaction with the non-polar portions of PLA and possibly a conformational change (exposing the polar groups on the surface of the fibers) [46].

### 2.2. Biocompatibility Test 

**Figure 5:**
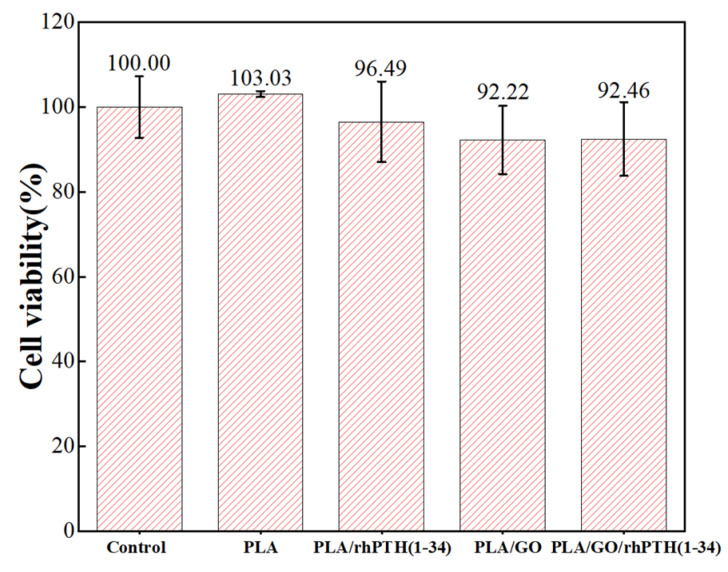
MTT assay of 2-day cell survival on the surfaces of composite membranes (Notes: *p* > 0.05 stands for no significant difference).

Although many studies have reported that low concentrations of GO or rhPTH(1-34) were biocompatible and well-used in the field of induced osteogenesis, it was still of great significance to conduct MTT experiments to observe whether the composite membranes were suitable for subsequent in vitro studies. In Figure 5, MTT analysis was performed to study the activity of MC3T3-E1 cells cultured on different membranes after 2 days. The cell viabilities of PLA, PLA/rhPTH(1-34), PLA/GO and PLA/GO/rhPTH(1-34) were 103.03 ± 0.71%, 96.49 ± 9.50%, 92.22 ± 8.03% and 92.46 ± 8.66%, respectively. Compared with the control group, there were no significant differences (*p* > 0.05), indicating that cells could maintain adhesion and survival on composite materials. According to these experimental results, PLA/GO/rhPTH(1-34) scaffolds had good biocompatibility.

**Figure 6:**
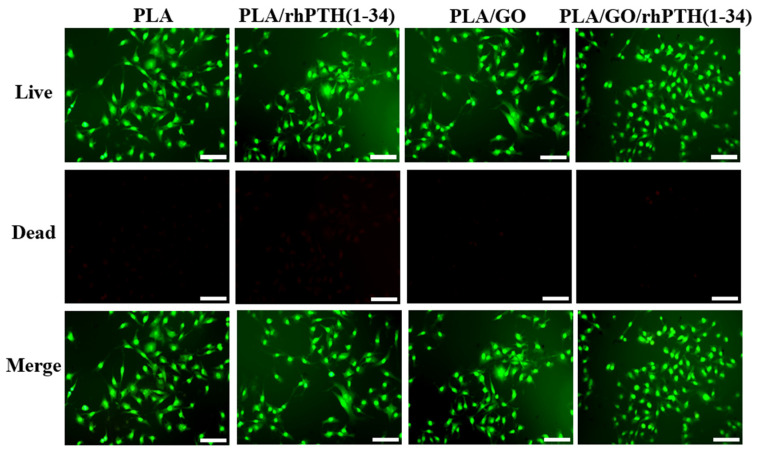
AO/EB staining assay of MC3T3-E1 cells cultured with various samples (100× magnification, Bar = 100 µm).

As shown in Figure 6, AO/EB double staining was performed using a fluorescence microscope to further evaluate the biocompatibility of various samples. The living cells were stained green by AO, whereas dead cells were stained red by EB. Based on this, it can be seen that the MC3T3-E1 cells cultured with as-prepared nanofiber membranes for 48 h had good viability, which indicated that PLA/GO/rhPTH(1-34) nanofiber membranes provided a suitable growth environment for MC3T3-E1 cells and supported cell proliferation, which was expected in a candidate material for bone tissue engineering.

**Figure 7:**
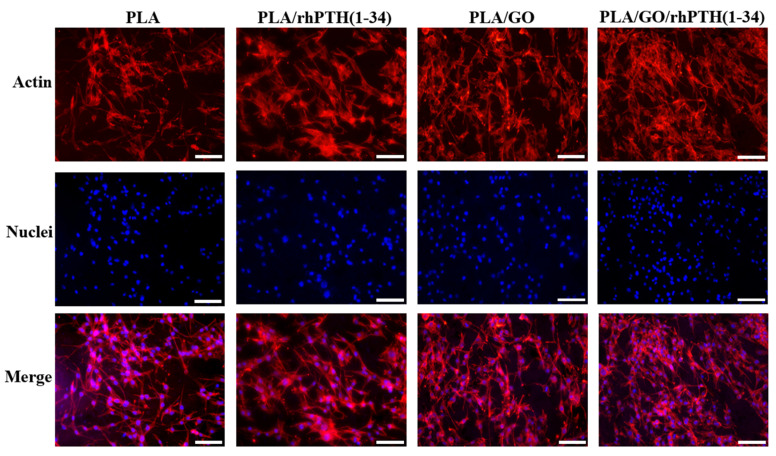
Cell adhesion and extension assay (100× magnification, Bar = 100 µm).

After incubation on the membrane for 48 h, the adhesion of cells was evaluated using fluorescence staining (Figure 7). The MC3T3-E1 cells in all groups showed a spreading and increasing trend. The cells attached to each sample showed similar morphology of MC3T3-E1 cells, the expression of actin could be clearly observed, and the cytoskeleton structures were neat. However, compared with the PLA group, the MC3T3-E1 cells in the other three groups presented elongated and diffused morphology, indicating good growth behavior of the cells. The results showed that the composite electrospun membranes had more advantages for cell adhesion and might have a positive effect on osteogenic differentiation during bone regeneration.

Ideal scaffold surfaces should support cell growth, and cell–biomaterial interactions play an important role in bone tissue engineering, where the biological behavior of cells is mainly influenced by surface morphology and chemical composition [47,48,49]. This study attempted to investigate the interaction between the polylactic acid membrane combined with two osteogenic materials and MC3T3-E1 cells. The results showed that the membranes containing GO and rhPTH(1-34) were more conducive to cell adhesion, and the cytoskeleton structures of the surfaces were regular. Therefore, the PLA/GO/rhPTH(1-34) membranes had good biocompatibility and no obvious cytotoxic effect on the MC3T3-E1 cells, showing their potential applications in bone tissue engineering.

### 2.3. Effect of Nanofiber Membranes on Osteogenic Differentiation 

**Figure 8:**
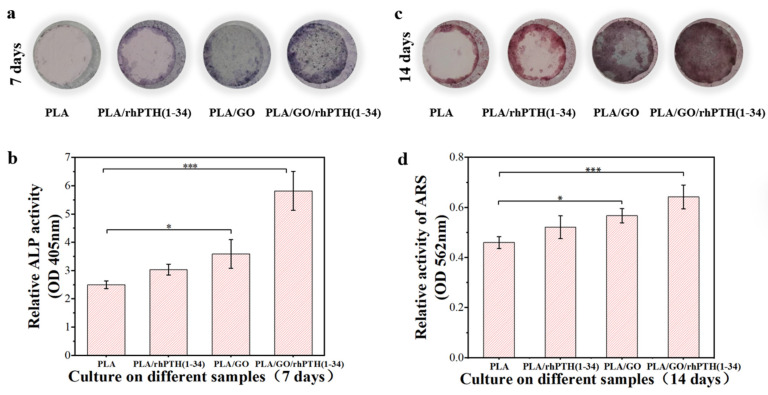
ALP activity and calcium deposition. (**a**) After 7 days of culture, MC3T3-E1 cells on the membranes were stained with ALP; (**b**) ALP activity was assayed using a quantitative colorimetric assay (Notes: * *p* < 0.05, *** *p* < 0.001); (**c**) after 14 days of culture, MC3T3-E1 cells on the different membranes were stained with ARS; (**d**) calcium deposition activity was assayed using a quantitative colorimetric assay (Notes: * *p* < 0.05, *** *p* < 0.001).

The ideal bone repair material should also enhance the osteogenic differentiation of cells [50]. In this study, the effects of electrospun membranes on the osteogenic differentiation of MC3T3-E1 cells were measured using ALP staining and calcium deposition. ALP is an important indicator of early expression in the process of osteogenic differentiation, and it has the ability to form the mineralization of the extracellular matrix [51]. By combining the results of ALP staining and quantitative detection (Figure 8a,b), the surface color of the PLA/GO/rhPTH(1-34) group was the deepest, and the expression of ALP on this group was significantly higher than that on others. The ARS and quantitative results were similar to those of the ALP staining (Figure 8c,d). In addition, osteoblast differentiation is controlled by a master transcription factor, Runx2, which is a key factor implicated early in the maturation of osteoblasts [52,53]. It has been reported that rhPTH(1-34) increased proteasomal proteolysis of Runx2, while inhibiting Runx2 degradation by E3 ligase [53]. It also had a dual effect on bone formation and resorption, and materials containing rhPTH(1-34) had better osteogenic effects on bone defects than materials without it, which is consistent with our experimental results. These results indicated that nano-GO powder exhibited good bone conductivity and promoted the proliferation of cells and bone regeneration, which was more obvious when it was combined with rhPTH(1-34).

The results obtained by our work suggest a feasible way to incorporate GO and rhPTH(1-43) into polylactic acid membranes using electrospinning technology, which forms a stable system to maintain the sustained release mode of local hormones. Studies have shown that this method improved the osteogenic activity of PLA, endowed PLA with good biocompatibility and further enhanced the osteogenic differentiation ability of MC3T3-E1 cells. Of course, further in vivo investigations are needed for clinical applications.

## 3. Materials and Methods

### 3.1. Materials

GO (>99%, powder) was purchased from Macklin Biochemical Co., Ltd. (Shanghai, China). RhPTH(1-34) (99.78%, powder) was purchased from Haoyuan Biomedical Technology Co., Ltd. (Shanghai, China). PLA pellets were purchased from Hisun Biomaterials Co., Ltd. (Revode190, Zhejiang, China). Trichloromethane (TCM, 98%) and N, N-dimethyl-formamide (DMF) were purchased from Sinopharm Chemical Reagent Co., Ltd. (Shanghai, China). Span 80 was purchased from Aladdin Scientific Co., Ltd. (Shanghai, China). The MTT assay kit and acridine orange/ethidium bromide were purchased from Solarbio Co., Ltd. (Beijing, China). A live/dead cell staining kit was purchased from UElandy Biotechnology Co., Ltd. (Suzhou, China). The TRITC-phalloidin staining solution and DAPI staining solution were purchased from Maokang Biotechnology Co., Ltd. (Shanghai, China). An ALP-Staining Kit was purchased from Beyotime Biotechnology Co., Ltd. (Shanghai, China). 

RhPTH (1-34) solution was prepared by dissolving 1 mg rhPTH (1-34) powder into 1 mL phosphate buffered saline (PBS, PH = 7.4), and then diluting to a final concentration of 10^− 7^ M.

### 3.2. Methods

#### 3.2.1. Fabrication of Electrospinning Fibers 

PLA/GO/rhPTH (1-34) composite fibers were prepared by the following methods: Firstly, 1.2 g PLA pellets were dissolved in 10 mL (TCM:DMF = 8:2) mixed solvent. Then, 0.05 g GO powder was dispersing into polymer under sonication for 10 min and stirred. After 24 h, 0.1 mL of 10^−7^ M rhPTH (1-34) and 0.03 mL Span 80 (a good w/o emulsifier) was added, stirred for 0.5 h to form a stable water-in-oil (w/o) emulsion. Finally, the solution containing GO (0.5 wt.%) and rhPTH (1-34) (10^−9^ M) was obtained. The mixed solution was drawn into a 5 mL plastic syringe connected to a needle. The electrospinning parameters were set to a 18–20 kV voltage range and the flow rate values were between 1.0 and 1.2 mL/h. An aluminum foil paper was used as the collector and placed 16–18 cm from the tip of the needle (SS-1334, YongkangLeye Technology Development Co., Ltd., Beijing, China). The process was carried out under controlled temperature (24.0 ± 0.5 °C) and relative humidity conditions. Finally, the samples were placed in a vacuum drying oven at 37 °C overnight to evaporate the remaining solvent.

PLA, PLA/GO and PLA/rhPTH (1-34) composite fibers were also prepared according to the above method and with the same concentrations of the corresponding materials.

#### 3.2.2. Characterization

The morphology of different electrospun membranes was observed through field emission scanning electron microscopy (FE-SEM), operating at an accelerating voltage of 5 kV (JEOL, JSM-6701F, Tokyo, Japan). All the samples were sputter-coated with a gold layer before the SEM observations. The diameter of the fibers was measured from the micrographs of 100 random fibers by using image analysis software (ImageJ, V1.8.0.), and the diameter distribution histograms were plotted by OriginLab software (2018). 

Fourier transform infrared (FTIR) analyses of electrospun fibers were performed using a Nicolet iS50 instrument in ATR mode from the range of 400 cm^−1^ to 4000 cm^−1^ with 64 scans.

The tensile properties of electrospun membranes were evaluated using a universal testing machine (Instron, CH17-621, applied load is 5 N) using ten samples from each group, with a 5 cm length × 0.5 cm width, 50 μm thickness of each membrane, at a crosshead separation speed of 5 mm/min. 

The contact angle analyzer (KINO, SL200KS, New York, NY, USA) was used to estimate the hydrophilicity of membranes. The contact angle was measured at 3 different locations on the membrane surface. A 3 × 0.5 cm membrane dimension and five replicates were tested to obtain the average values.

#### 3.2.3. Biocompatibility Test

##### Cell Activity

The samples used in the cell test were sterilized using UV exposure. First, the MC3T3-E1 cell line viability was measured using the MTT analysis. In a 96-well plate, cells (100 μL, 8 × 10^4^ cells/mL) were seeded on 6-mm diameter membranes and incubated for 2 days at 37 °C with 5% CO_2_. After the incubation was completed, 150 μL of fresh medium mixed with MTT reagent (5 mg/mL, PBS) was added to each well and incubated in the dark for 4 h. Then, the MTT and residual medium in the well plate were removed, and 150 μL of DMSO was added to dissolve formazan. Finally, the solution from each experimental well-plate was removed to the blank plates, and optical density (OD) was measured at 490 nm using a microplate reader to assess the viability of cells.

##### AO/EB Staining

MC3T3-E1 cells (100 μL, 8 × 10^4^ cells/mL) in incubation medium were seeded on sterile electrospun membranes in 96-well plates under the same incubation conditions for 48 h. The incubation medium was removed, and then 100 μL of the acridine orange/ethidium bromide (AO/EB) mixture was added in the dark for 15 min. Finally, the cells were washed with PBS twice to remove the unreacted stain and the survival of cells was observed using fluorescence microscopy.

##### Cell Adhesion and Extension

MC3T3-E1 cells (100 μL, 4 × 10^4^ cells/mL) were seeded on sterile electrospun membranes in 96-well plates under the same incubation conditions for 48 h. After 48 h of cultivation, the cells were fixed with 4% paraformaldehyde (PFA) for 10 min and treated with 0.5% Triton X-100 for 5 min. The cytoskeleton was stained with TRITC-Phalloidin, and the nuclei were stained with 40, 6-diamidino-2-phenylindole (DAPI, 100 nM) for 1 min, observed and photographed under a fluorescence microscope.

#### 3.2.4. Osteogenic Differentiation

##### Alkaline Phosphatase (ALP) Activity Assay and Calcium Deposition

After 7 days of inoculation on the surfaces of the different samples, cells (100 μL, 4 × 10^4^ cells/mL) were fixed using 4% PFA, and a BCIP/NBT alkaline phosphatase color development kit (Beyotime, Shanghai, China) was added for staining for 20 h at 37 °C. The samples were rinsed twice with deionized water, dried, and photographed under a microscope. For the quantitative ALP assay, the cell lysates were incubated with p-nitrophenyl phosphate (pNPP, Beyotime, Shanghai, China) at 37 °C for 30 min. ALP activity was tested by detecting the optical density (OD) values at 405 nm. 

After 14 days of inoculation on the surfaces of the different samples, the cells (100 μL, 4 × 10^4^ cells/mL) on the membranes were fixed using 4% PFA and stained using 0.1% alizarin red S (ARS). The samples were rinsed twice with deionized water, dried and photographed. For the quantitative assay, the stained cells were desorbed with 10% cetylpyridinium chloride (Sigma, St. Louis, MO, USA), and the OD values were detected at 562 nm. 

#### 3.2.5. Statistical Analysis 

All the experiments were independently repeated at least three times. Results were presented as the mean ± standard deviation. The statistical analysis was performed using the GraphPad Prism statistical software(V8.0.2). One-way ANOVA was performed, followed by Dunnett’s test for multiple comparisons (* *p* < 0.05, ** *p* < 0.01, and *** *p* < 0.001).

## 4. Conclusions

In this study, GO and rhPTH (1-34) were blended with PLA via an electrospinning technique to prepare functional PLA nanocomposites. The tensile strength and elastic modulus of PLA/GO/rhPTH(1-34) membranes increased to 2.6 MPa and 34.0 MPa, respectively, which are much higher than those of pure PLA. The in vitro experiments showed that the bioactive components of GO and rhPTH(1-34) can upregulate the alkaline phosphatase activity, and increase the calcium deposition. After 7 and 14 days of culture with MC3T3-E1 cells, a quantitative test of the PLA/GO/rhPTH(1-34) membranes showed a 2.3-fold increase in the ALP activity and a 1.5-fold increase in calcium deposition. Therefore, the assumption that mechanical properties and osteogenic differentiation ability can be improved through the use of PLA/GO/rhPTH(1-34) composite membrane scaffolds is correct. The scaffolds may have good potential for bone tissue regeneration and provide a new approach for the application of graphene and hormone materials in bone tissue engineering.

## Data Availability

The data used in this work are available from the first authors or the corresponding authors.
